# Keystone Veterinary Medical Association

**Published:** 1896-12

**Authors:** W. L. Rhoads

**Affiliations:** Secretary


### SOCIETY PROCEEDINGS.

### KEYSTONE VETERINARY MEDICAL ASSOCIATION.

WHAT proved to be one of the most pleasant and instructive meetings ever held by the Association was called to order by President John R. Hart, Tuesday evening, October 13th, with the following members of the profession present: Drs. Francis S. Allen, H. A. Christman, C. M. Cullen, Charles T. Goentner, John R. Hart, W. Horace Hoskins, Charles Lintz, Thomas B. Rayner, and W. L. Rhoads.

After the reading and approval of the report of the September minutes, the Chairman of the Committee on Certificates reported the progress of a most diligent search for the original minute-book of the Association that they might have the correct date of its organization placed on the membership certificates.

Dr. H. J. McClellan, having given up the profession of veterinary medicine, tendered his resignation to the Association, which was read and referred to the Board of Trustees.

Dr. Hoskins then read an interesting paper entitled "Some Aspects of Association Works and Aims," in which he urged the necessity of a closer union of the profession at this time when we are so ill-prepared to stand alone as individuals; further than this, we need to foster a closer affiliation with our brother profession, that of human medicine. He spoke of the great danger to our own welfare and prosperity by the establishment in our midst of great marts of trade toward which every channel of trade seems to run. As a rule, their influences are not for the general good. He cited an instance where there had been concentrated under one roof the business of fifty or more individual houses, thus losing to the community the value of a number of good and efficient men. To the acute observer this means a loss to the manufacture of drugs and chemicals, as well as the retailer and dispenser, also the carriage-builder, the harness-maker, the shoer, etc., *ad finitem.* Undoubtedly the strength of our Association circles first brought before the people the advantage of civil-service reform, and, after thoroughly enthusing them on the subject, has year by year gradually raised the standard of such examination till now, not the politician, but the proficient professional man is most sure of his position. Thus the Association is an incentive to all to keep moving toward the top. The reading of this paper, which, by the way, was up to date, was listened to with great interest, and evidently thoroughly digested by each one fortunate enough to hear it.

The first of the half-hour talks arranged for this season's entertainment was by Dr. J. Cheston Morris, "On Some Statistics of Dairy-products and Thoughts Thereupon; Personal Experience in the Proper Preparation of Milk for Sale in the Markets and the Value of Separator-products of Milk." This, though looked forward to with great interest, was to all a most delightful surprise, as it was, without doubt, the most thoroughly comprehensive and instructive talk on this or kindred subjects ever given before the Association. Dr. Morris spoke of the superiority of Devon cattle for the production of milk to be used in its natural state, as due to its relative proportions of butter-fat, casein, and sugar being the same, or nearly so, as those of human milk, as shown by the many thousands of analyses made and published by the Aylesbury Dairy Company and the society of chemists in Great Britain; from these we may gather that Jerseys and Guernseys yield a milk relatively rich in fat, but poor in casein and sugar. Short-horns, Ayrshires, and Holsteins a more watery fluid, rich in casein, but relatively poor in butter-fat and sugar; while Devons give milk nearly as rich in fat as the Jersey, and in casein as the Ayrshires, and richer than either in sugar. While the fat in a finely-divided natural emulsion is most useful and necessary in cell-growth, we are apt to overvalue it and disregard the active agency of milk sugar, which, by its splitting into lactic acid in the digestive processes, causes the chemical changes in the phosphates and albuminates on which tissue-building and nutrition so largely depend. Hence the superiority of Devon milk, as shown in practical results. Skimmed milk yields a hard curd, difficult of digestion. The curd of cow's milk being more dense than the light, flocculent curd of human milk, in artificial feeding we use sugar or starchy material to make the curd more porous or spongy. The milk of the buffalo, goat, and ass illustrates the same truth. Lehman says a cow properly fed should yield 1 per cent. of her weight. The statistics kept by Dr. Morris (and they are certainly most complete and exhaustive, each sheet showing name, herd-book number, when dropped, when she calved, sex, color, and any peculiarity of calf, what became of it, if kept or sent to the butcher, when she comes in profit, the number of pounds of milk given each night and morning, when she is served and when due, her price and any peculiarities of her conformation, etc.; these sheets, being carefully filed, give a complete life-history of each cow and her offspring) show that his cattle, which weigh from six to eight hundred pounds, have averaged him five-and-a-half times their weight, or about 3500 pounds of milk yearly (a most conservative estimate), on a feed of hay, corn-fodder cut green and mixed with hominy, bran, potatoes, and beets. He explained the method of the Devonshire people for raising cream and making butter, the sweet skimmed milk being fed to the calves. He has tried this process, but is now using a hand-separator, with which recently he obtained over four quarts of cream from twenty-eight quarts of milk (one pound of butter from seven quarts of milk), which he can use more profitably for calves than for pigs. After the talk Dr. Morris expressed a willingness to answer any questions put to him. He was kept busy for the next half-hour, being questioned relative to almost every stage and era of dairy-breeding and farming. Yet in every instance he was fully prepared, showing his great interest in and love for this work. In answer to some of these he described Guenon's system for selecting milkers by their markings—known as the scutcheon. He is in favor of inspection not only of the cattle, but of attendants and those who handle milk, claiming a tuberculous milkman may do more harm than a great number of tuberculous cattle. This examination should extend not only to the health of the cattle, but to their cleanliness and care of stables, milk-houses, etc. This examination should be made frequently and without warning. He felt the general average of health might be improved by proper food; also that the Danish method of separating tuberculous cows and using their milk after boiling to raise their own calves has been shown to be perfectly safe and successful, thus rendering wholesale slaughter unnecessary, and might be done without entailing a great loss. He figures it now costs four cents per quart to raise milk with hay at fifteen dollars per ton.

Dr. Rhoads now moved that in recognition of the great value of this talk to those present, and in consideration of the benefit his attendance and fellowship would be to us, that he be made an honorary member; this was seconded by Dr. Hoskins, and unanimously voted upon. The hour being late, election of officers was postponed till the November meeting.

W. L. Rhoads, D.V.S.,
Secretary.

